# Human Olfactory Receptor Sensor for Odor Reconstitution

**DOI:** 10.3390/s23136164

**Published:** 2023-07-05

**Authors:** Shun’ichi Kuroda, Yukiko Nakaya-Kishi, Kenji Tatematsu, Shuji Hinuma

**Affiliations:** 1 Department of Biomolecular Science and Reaction, SANKEN, Osaka University, 8-1 Mihogaoka, Ibaraki, Osaka 567-0047, Japan; kenji44@sanken.osaka-u.ac.jp (K.T.); hinuma@sanken.osaka-u.ac.jp (S.H.); 2 R&D Center, Komi-Hakko Corp, 3F Osaka University Technoalliance C Bldg, 2-8 Yamadaoka, Suita, Osaka 565-0871, Japan; kishi@komi-hakko.co.jp

**Keywords:** olfactory receptor, odor sensor, cell array sensor, odor matrix, odor matrix library

## Abstract

Among the five human senses, light, sound, and force perceived by the eye, ear, and skin, respectively are physical phenomena, and therefore can be easily measured and expressed as objective, univocal, and simple digital data with physical quantity. However, as taste and odor molecules perceived by the tongue and nose are chemical phenomena, it has been difficult to express them as objective and univocal digital data, since no reference chemicals can be defined. Therefore, while the recording, saving, transmitting to remote locations, and replaying of human visual, auditory, and tactile information as digital data in digital devices have been realized (this series of data flow is defined as DX (digital transformation) in this review), the DX of human taste and odor information is not yet in the realization stage. Particularly, since there are at least 400,000 types of odor molecules and an infinite number of complex odors that are mixtures of these molecules, it has been considered extremely difficult to realize “human olfactory DX” by converting all odors perceived by human olfaction into digital data. In this review, we discuss the current status and future prospects of the development of “human olfactory DX”, which we believe can be realized by utilizing odor sensors that employ the olfactory receptors (ORs) that support human olfaction as sensing molecules (i.e., human OR sensor).

## 1. Introduction

**Figure 1:**
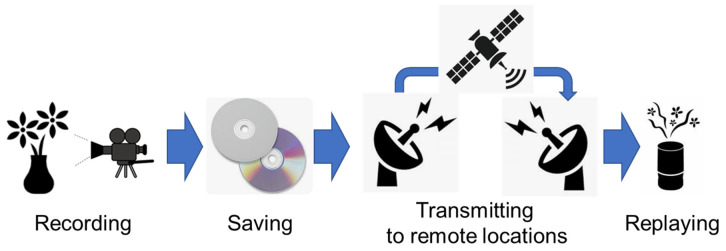
Conceptual diagram of human olfactory DX. All gas molecules (simple or complex odors) recognizable by humans are measured with sensors (represented by a pictogram of a camera) that have the same odor discrimination ability as the human olfactory system (recording). The data can be recorded as univocal digital data in a simple common format, saved in a portable memory device (saving), and transmitted to remote locations through radio waves or internet (transmitting to remote locations). The original odor can be reconstituted based on the data by a diffuser capable of mixing odor molecules in real time (replaying).

**Figure 2:**
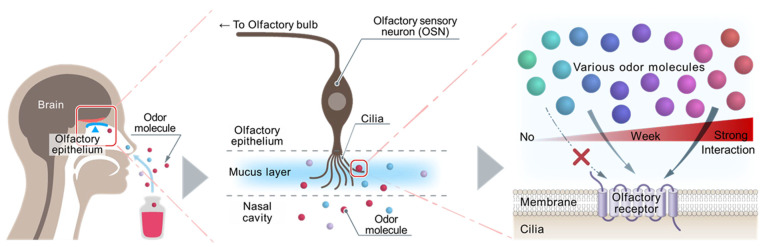
Olfactory sensory neuron (OSN) and olfactory receptor (OR) in the human olfactory epithelium. Odor molecules entering the nasal cavity are recognized by 388 types of OR on OSN in the olfactory epithelium (**Left**). Each OSN expresses a different OR in the cilia extending to the mucus layer, enabling it to bind to odor molecules. OSN can transduce to the olfactory bulb the membrane potential change induced by odor molecules binding to the OR (**Middle**). When an odor (simple or complex) encounters the entire 388 ORs, and the contained various odor molecules bind to some OR groups with different affinities, pattern recognition of the entire odor by the 388 ORs is performed (**Right**). The human olfactory system can discriminate huge varieties of odors by this mechanism.

For implementing “recording, saving, transmitting to remote locations, and replaying” of human olfactory information (defined as human olfactory DX; Figure 1) in the next generation of information devices, it is necessary to represent all odors (whether simple or complex) perceived by human olfaction as objective and univocal digital data in a simple common format as much as possible. If human olfactory DX is realized, people around the world will be able to share the same odor in real time, which will revolutionize means of expression by introducing olfactory information to the existing visual and entertainment industries, as well as the metaverse in XR (extended reality), which has until now relied solely on visual and auditory information. The conventional odor-quality-evaluating methods used for this purpose can be classified into three main categories: (1) metal-oxide-based or organic-polymer-based semiconductor sensors that can only detect a limited number of odor molecules and no matter how many of these sensors are combined, cannot cover all odors that humans perceive in principle, (2) GC–MS (gas chromatography–mass spectrometry) that detects all gas molecules, even those that humans cannot detect; its output data are often complicated and difficult to convert into simple digital data, and (3) sensory tests that depend on each individual’s olfactory characteristics. This means that these three methods are not necessarily sufficient for human olfactory DX realization. Other sensors have been recently developed that utilize biological sensing molecules (e.g., odorant-binding proteins (OBPs) [1,2], OBP-derived peptides [3], insect olfactory receptors (ORs) [4]) and have shown high sensitivity and discriminatory ability for specific odor categories. These sensors, however, cannot contribute to the realization of human olfactory DX unless they are able to detect and discriminate all odors (simple or complex) perceived by the human olfactory system. In human olfaction, ORs (the number of all human ORs is defined as 388 in this review) are expressed on olfactory sensory neurons (OSNs) in the olfactory epithelium. Each OR is activated at different intensities for each odor molecule, and human olfaction recognizes odors with an overall activation pattern [5] (Figure 2). It is thought that if the activation intensity of each OR is used as an index, almost all odors (simple and complex) perceived by human olfaction will be capable of representing 388 dimensional parameters. This means that if all human ORs are used as sensing molecules, almost all odors that humans perceive can be detected and discriminated, which is essential for human olfactory DX realization. All the odor evaluating methods mentioned above cannot be used for DX realization because they either detect only some limited odors, detect gas molecules that do not smell, cannot discriminate complex odors, or are not objective methods. At present, sensors that use all human ORs as sensing molecules are the only way to realize human olfactory DX.

## 2. Establishment of Heterologous Cells Expressing ORs

**Figure 3:**
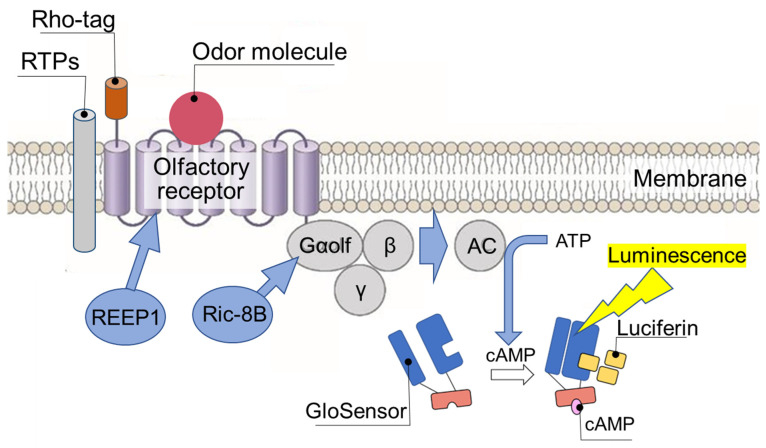
Endpoint measurement of the binding affinity of odor molecules to OR expressed on heterologous cells. To promote OR expression on the surface of heterologous cells, a signal peptide (Rho-tag) is added to the *N*-terminus and the OR chaperones RTPs (RTP1, RTP1S, RTP2) and REEP1 and Gα protein chaperone Ric-8B are coexpressed. Binding of the odor molecule causes OR to activate adenylate cyclase (AC) via trimeric G proteins to produce cAMP. The amount of cAMP is assayed by endpoint measurement of chemiluminescence using GloSensor^TM^ and is determined as the binding affinity between the odor molecules and OR.

Many attempts have been made so far to identify OR groups that respond to any odor molecule by using OSNs isolated from mouse olfactory epithelium (i.e., deorphanization). Recently, a robot with built-in time-lapse single-cell array cytometry has been developed to automatically pick up only one OSN that responds to odor molecules from the cell array containing mouse olfactory-epithelium-derived cells [6], but it is still necessary to isolate olfactory epithelium from mouse neonates with complicated procedures. Furthermore, it is ethically difficult to isolate human olfactory epithelium. Thus, the recent major method is to create a library of all human OR-expressing cells by recombinant DNA technology and screen odor-responsive OR-expressing cells by using any given odor [7]. However, when ORs are expressed in heterologous cells, they often aggregate and accumulate in the endoplasmic reticulum (ER) and are degraded [8], making it difficult to express all human ORs on the cell surface and enable them to respond to odor molecules. Matsunami et al. found that human embryonic kidney-derived HEK293 cells introduced with the chaperones RTP1 (receptor-transporting protein 1), RTP2, and REEP1 (receptor expression-enhancing protein 1), also called Hana3A cells, greatly improved cell surface expression of ORs with an *N*-terminal Rho-tag (rhodopsin-derived signal peptide) [9] (Figure 3). In particular, RTP1S, a C-terminal part shortened RTP1, more strongly improved the cell surface expression of ORs and odor molecule responses [10]. The Lucy-tag [11] and the IL-6-Halo-tag [12] also enabled cell surface expression of a wider range of ORs than the Rho-tag. Furthermore, co-expression of non-OR GPCRs (G protein-coupled receptors) (e.g., β2-adrenergic receptor, M3 muscarinic acetylcholine receptor) formed heterodimers with ORs and improved their sorting to the cell surface [13,14]. Co-expression of M3 muscarinic acetylcholine receptor has been found to suppress β-arrestin 2-mediated OR internalization [15,16]. Other improvements in second messenger (cAMP) generation and detection systems have also been achieved through the co-expression of olfactory-specific G protein α GNAL/Gαolf [17], which has high affinity for ORs, Ric-8B [18], a chaperone of Gα protein, and GloSensor^TM^ [19], a highly sensitive luciferase for cAMP detection. The above improvements have made the majority of human ORs presentable on the cell surface of HEK293 cells, increasing the likelihood of identifying a group of ORs that respond to any given odor molecule [20]. More recently, common structural features of ORs that are expressed on the cell surface independent of RTPs have been found [21,22]. Introducing mutations in all these structural features into all ORs will likely result in their successful expression on the cell surface. However, since the mutational sites are distributed throughout the OR molecules, it is likely that the original odor molecule recognition mechanism will be altered. Thus, these mutant ORs unfortunately cannot be used for comprehensive deorphanization for human olfactory DX realization.

## 3. Importance of Measuring OR Response in Real Time

The attempts to improve cell surface expression and odor molecule responsiveness of ORs as described in Section 2 are very important, but most of these improved cells have been used exclusively for the endpoint measurements of cAMP generation after prolonged odor stimulation (30 min to several days). Continuous exposure of odor to OR-expressing cells for a long period of time should be avoided because some odor molecules are easily denatured even at room temperature (e.g., oxidation, hydrolysis) or cytotoxic; thereby, OR responses to these odor molecules will be different from the original OR responses.

In addition, human olfaction responds immediately after odor stimulation and adapts within a few minutes. This process over time is highly dependent on the OSN’s OR response to odor. While the best way to realize human olfactory DX is to measure OSN action potential changes in real time, at present it is difficult to measure all membrane potential changes in many OR-expressing cells at once in real time. Therefore, as a practical method, it is important to measure intracellular Ca^2+^ influx in real time, since CNG (cyclic nucleotide-activated channel) induces membrane potential changes in OSNs by mobilizing Ca^2+^ ion into the cell simultaneously with Na^+^ ion [23].

## 4. Human Olfactory Receptor-Expressing Cell Array Sensor (Human OR Sensor)

**Figure 4:**
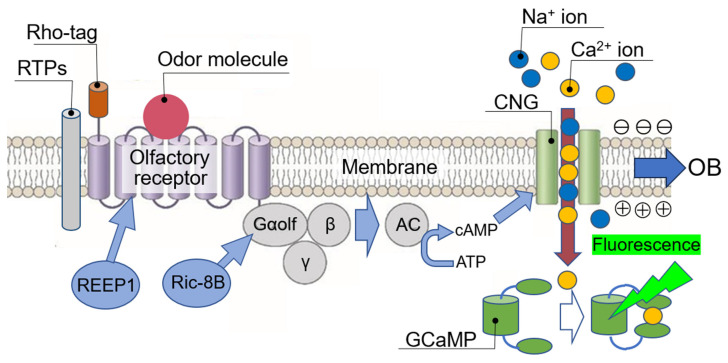
Real-time measurement of the binding affinity of odor molecule to OR expressed on heterologous cells. Binding of odor molecule causes OR to activate adenylate cyclase (AC) via trimeric G proteins to produce cAMP, which activates and opens cyclic nucleotide-activated channels (CNGs) to promote intracellular influx of Na^+^ ion and Ca^2+^ ion, resulting in the induction of a membrane potential change. In the human olfactory system, this transient membrane potential change is transmitted from the OSN to the olfactory bulb (OB) as part of the human olfactory information. Real-time changes in the amount of intracellular Ca^2+^ influx are measured by the fluorescence intensity change of Ca^2+^-dependent fluorescent protein (GCaMP) and are determined as real-time changes in the binding affinity between odor molecules and OR.

**Figure 5:**
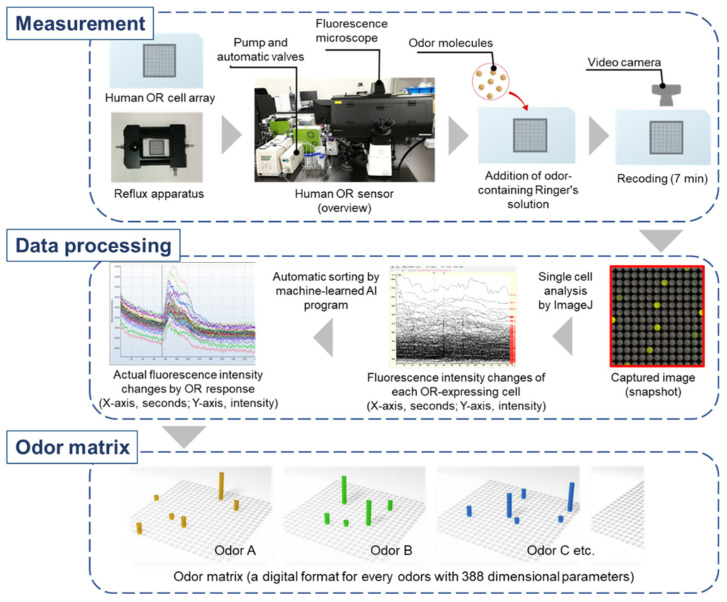
Workflow from odor measurement with human OR sensor, single cell-based analysis of fluorescence intensity changes, and odor matrix generation. Note that these odor matrixes also include changes over time for each OR; for easier understanding, a snapshot of the time when the fluorescence intensity reached its maximum is usually used.

For comprehensive measurement of the real-time response of all human ORs to various odor molecules simultaneously, CNGs and Ca^2+^-dependent fluorescent protein (GCaMP) were stably expressed in HEK293T cells optimized for OR expression as described in Section 2, followed by expression of each of the 388 human ORs [24] (Figure 4). Next, 0.5 mm square microwells were printed in 20 rows by 20 columns on a glass slide (total 400 microwells) with hydrophobic ink, and a dispensing robot was used to make a cell array with approximately 400–500 cells of each OR-expressing cell in each microwell. After 48 h incubation, the cell array was set in a reflux apparatus under a fluorescence microscope equipped with a video camera. This system for simultaneous and real-time measurement of the response of all human ORs to odor is called a human olfactory receptor-expressing cell array sensor (hereafter abbreviated as human OR sensor; Figure 5). Ringer’s solution was used in dissolving the odor of interest whether simple or complex; the concentration of odor molecules used in the measurements, unless otherwise noted, is as follows: 0.01–0.1 mM for simple odor molecules, and 0.05–5.0 mM for complex odor molecules, was refluxed continuously. Immediately after the odor molecules reach the cells, cells expressing ORs in response to the odor molecules begin to emit fluorescence. For 7 min, a fluorescent image of the entire array was captured on video to record the fluorescence emitted by cells expressing ORs responsive to the odor molecules (0.67 fps (frame per seconds); 16 bits; 5.0 × 10^6^ pixels; data size of about 6 GB; tiff (tag image file format) file). Then, all fluorescence intensity changes were datamined at a single-cell basis (four pixels per cell) by ImageJ program ver. 1.52u (image processing and analysis in Java; Wayne Rasband, National Institute of Health, Bethesda, MD, USA). Fluorescence intensity changes of each cell that varied by 5% or more were defined as significant, based on the fold factor of the fluorescence intensity after odor addition relative to the average fluorescence intensity for 10 s before odor addition. Each cell emits spontaneously numerous nonspecific fluorescence due to the intracellular Ca^2+^ mobilization by normal cellular activity. Therefore, using a machine learning AI program to process a large number of temporal changes in fluorescence emitted by cells expressing odor-responded ORs, the actual changes in fluorescence intensity due to odor-responded ORs were extracted and stored as CSV (comma-separated values) files. Dozens of the actual changes in fluorescence intensity per OR were extracted and averaged. By this method, variation in fluorescence intensity changes due to biological fluctuations in OR-expressing cells could be suppressed to around 10% CV (coefficient of variation). Various odors, both simple and complex, can be represented by the response intensity of 388 human ORs (called the odor matrix; see Figure 5). Note that this odor matrix also includes changes over time for each OR, since the same OR showed different changes in fluorescence intensity for different odor molecules [24]. For easier understanding, a snapshot of the time when the fluorescence intensity reached its maximum is usually used. Although a similar real-time measurement of OR response can be performed with FLIPR (fluorescent imaging plate reader) [25], which is used by many pharmaceutical companies to screen GPCR agonists and antagonists, this human OR sensor based on cell array technology does not require many microplates or reagents, making it much faster and cheaper to measure different conditions sequentially without replacing the cell array sensor in the same measurement set.

## 5. Towards Human Olfactory DX Realization

Comprehensive deorphanization to determine exactly how all ORs recognize various odors in human olfaction remains challenging for current human OR-expressing cells. First, when the Gα protein in the cells used for OR expression is different from the OSN, the odor molecular response of some ORs may be altered [26]. Second, single-nucleotide polymorphism (SNP) is found in many human OR genes, which sometimes alters the odor molecular response of OR [27,28,29]. Third, almost all odor sensors using OR-expressing cells measure the OR response to odors in the liquid phase. In human olfaction, the ORs of OSNs respond to odors in the gas phase via a very small amount of nasal discharge, resulting in the detection threshold being much lower (typically 100- to 1000-fold) compared to the OR-based sensors. Currently, we cannot correctly explain this large difference in OR detection thresholds for odors. Indeed, these three issues are major obstacles to unraveling the scientific proposition of how the entire human ORs discriminates various odors with high sensitivity. However, for realizing human olfactory DX (especially in the processes of recording, saving and transmitting to remote locations), an odor sensor does not necessary perfectly reproduce the way human olfaction perceives odors. It is sufficient to always measure odors with a representative OR set (i.e., 388 ORs) under fixed conditions and define them in a 388-dimensional odor matrix.

## 6. Preliminary Odor Reconstitution

So far, the original odor has been reconstituted by analyzing its components by GC-MS and mixing the major odor molecules in the same proportions. However, odor molecules that cannot be detected by GC-MS often contribute significantly to odor quality, so perfumers had to spend a lot of time for formulation with many odor molecules by trial and error. For the achievement of the reconstitution part of the human olfactory DX, this process would be virtually impossible without preparing in advance all the approximately 400,000 types of odor molecules that exist in the world [30]. In other words, it is imperative to reduce the number of odor molecules used for the odor reconstitution of human olfactory DX. There is an attempt to reduce the number of odor molecules by GC-MS analysis of essential oils to sort out odor molecules that exhibit the same odor quality and reconstitute the odor quality of any given essential oil [31], but this method is applicable only to specific categories of odors (e.g., essential oils) and cannot be used to reconstitute a wide range of all odors.

**Figure 6:**
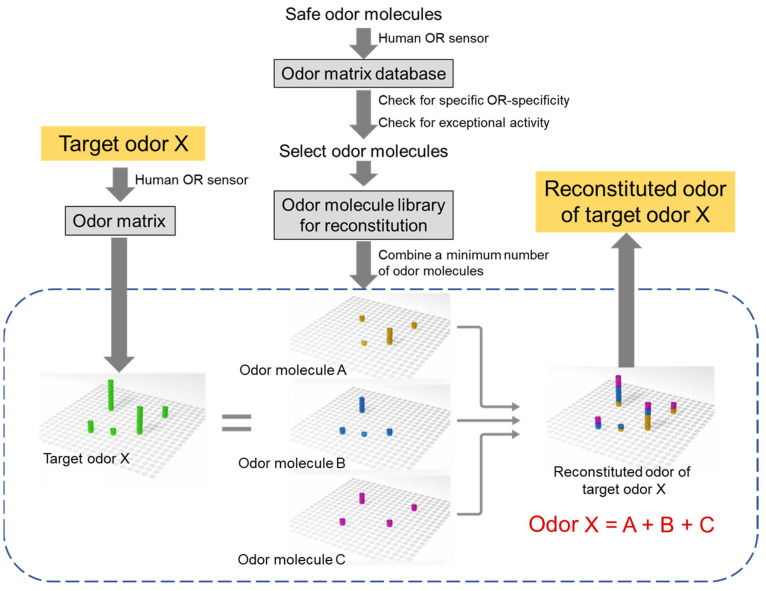
Conceptual diagram of odor reconstitution. First, a lot of basically safe odor molecules (e.g., natural flavors and fragrances, synthetic fragrances, food additives) are measured by a human OR sensor to build an “odor matrix database”. Incorporate odor molecules that selectively stimulate limited numbers of ORs in the database, exclude odor molecules showing exceptional activities described in Section 6 from the database, and generate an “odor molecule library for reconstitution”. The minimum group of odor molecules necessary to reproduce this target odor matrix (including changes in each human OR response over time) is selected from the library. As a result, in fragrance and flavor development, aromas and flavors with difficult-to-obtain or complex compositions can be reconstituted with odor molecules of easy-to-obtain or simple compositions. Furthermore, people around the world will be able to share the same odor in real time, which will revolutionize the expression method by introducing olfactory information to the existing visual and entertainment industries, as well as the metaverse in XR, which has until now relied solely on visual and auditory information.

Thus, by taking advantage of human OR sensors, we preliminarily reconstituted the odors of dried bonito flakes, rose essential oil, lavender essential oil, and vanilla flavor. Even though each sample was found to contain numerous types of odor molecules (>500, >50, >50, and >50, respectively) by GC-MS, 10 human ORs responded significantly to dried bonito flakes, 4 ORs to rose essential oil, 3 ORs to lavender essential oil, and 7 ORs to vanilla flavor. In addition, several of the responsive ORs were common. Therefore, odor molecules that selectively stimulate these ORs were selected from the odor molecule library and mixed in a ratio that could reproduce the odor matrix. Finally, the odors of dried bonito flakes, rose essential oil, lavender essential oil, and vanilla flavor were reconstituted with seven, four, three, and five odor molecules that were not included in the respective original samples, for which the odor quality was determined by sensory tests on a scale of several dozen people to be nearly reproduced [32]. This result strongly suggested that no matter how complex an odor is, if an odor matrix is obtained by a human OR sensor and mixed with odor molecules that stimulate each response OR with pinpoint accuracy, it is possible to reconstitute the odor with far fewer types of odor molecules (Figure 6).

In general, the OSN response to complex odors is essentially a linear relationship with the OSN response of each odor molecule [33]. Nevertheless, there is also intermolecular interference between odor molecules when multiple odor molecules bind to an OR [34]. This competitive binding result in a nonlinear relationship between the OR responses of multiple odor molecules [35]. These are also explained by a model in which odor molecules bind to allosteric pockets on the OR. In addition, there are inverse agonists in which the same odor molecules promote or inhibit OR responses in a concentration-dependent manner [36]. Human OR sensors capable of measuring even complex odors can detect odor molecules eliciting these exceptional OR responses. Only odor molecules that selectively stimulate limited number of ORs without exceptional activities in ORs should be used to reconstitute odors that have almost the same odor quality as any odor.

## 7. Future Improvements of Human OR Sensor

The main issue with human OR sensors is that their detection threshold for odor molecules is higher than that of human olfaction. To ameliorate this issue, we should consider suppressing mechanisms that inhibit intracellular cAMP generation (PDE (phosphodiesterase)) and intracellular Ca^2+^ mobilization (CaMKII (Ca^2+^-calmodulin-dependent protein kinase II), CaM (calmodulin), NCX (Na^+^/Ca^2+^ exchanger), PMCA (plasma membrane calcium pump)) [37,38,39], utilize extracellular OBPs that promote odor molecule presentation to the ORs [40], utilize intracellular OMPs (olfactory marker proteins) that increase intracellular cAMP levels [41,42], and utilize GRK2 (G-protein-coupled receptor kinase 2) inhibitors that suppress the binding of β-arrestins that promote OR internalization [43].

Recently, as there have been some reports that conventional ORs are not enough to detect all odors, human OR sensors should also be equipped with OSN-specific TAARs (trace amine-associated receptors; TAAR1, TAAR2, TAAR5, TAAR6, TAAR8, TAAR9) [44,45,46] to detect amine compounds and TRPs (transient receptor potential channels; 6 subfamily, 27 TRPs) [47,48] to detect odor molecules such as capsaicin and menthol. Since the second messengers of TAARs and TRPs are Ca^2+^ ions, the degree of activation of both TAARs and TRPs can be measured by simply diverting the cells used in the OR-expressing cells of human OR sensors.

The sensitivity of human olfaction is known to decline with age [49], but it is not a problem for daily life. This fact suggests that we should question if all ORs are necessary or not. Recently, not all OR mRNAs have been found in human olfactory epithelium by RNAseq analysis [50]. All OR mRNAs were not equally expressed and 26 major OR mRNAs accounted for more than 90% of the total OR mRNAs [51]. These facts indicate that the number of ORs in current human OR sensors may be overrepresented.

## 8. Possible Alternatives for Human OR Sensor

So far, it has been time consuming and labor intensive to perform comprehensive deorphanization of all human ORs by using conventional OR-based sensors; thus, OR databases (ODRactor, OlfactionBase DB) have been built to predict which ORs respond to any given odor molecules from OR structures [52,53,54]. Recently, deorphanization has been made possible by using a machine-learned proteochemometric model based on the structure of odor molecules and the orthosteric pocket sequences of responded ORs [55]. In addition, an AI that learned the experimental results of interactions between odor molecules and OR structures succeeded in predicting the responsive ORs to any given odor molecules with a correct response rate of over 90% [56]. These predictions are promising, but they need to be biologically validated in any case. In the future, these methods will certainly complement the human OR sensor method for human OR deorphanization.

Finally, because human OR sensors utilize living cells, odor measurement must always be performed in the laboratory after the sample has been collected elsewhere. Thus, it is not possible to use human OR sensors easily and readily in all settings. Recently, electrical odor sensors using only human OR proteins as the sensing molecules have been experimentally fabricated as eNose, which will compensate for the low portability of the human OR sensor [57]. For example, FET (field effect transistor) sensors in which liposome-based mini-cells incorporating ORs, adenylate cyclase, and CNG are immobilized via CNT (carbon nanotube) [58], multichannel FET sensors in which multiple ORs are directly immobilized via CNTs [59], and FET sensors immobilized with ORs embedded in a lipid membrane (nanodisc) [60] have been developed. These FET sensors using only human OR proteins surprisingly showed very low detection thresholds for odor molecules, comparable to human olfaction. This fact strongly suggests that the high detection threshold of the current human OR sensors is not caused by the OR molecules themselves, but by the intracellular signal transduction mechanism elicited by the ORs. These FET sensors, albeit highly sensitive, only detect interactions between odor molecules and ORs, and it may be difficult to correlate them with the odor matrix, which reflects the temporal changes of second messengers inside OSNs.

## 9. Conclusions

The emergence of human OR sensors has largely solved the technical issues that hampered the realization of human olfactory DX, though not sufficiently for a comprehensive understanding of the odor recognition mechanism in human olfaction. From now on, it is necessary to build up an “odor matrix database” of a huge number of odors while improving human OR sensors (Figure 6). In this process, instead of randomly measuring odors everywhere, the odor molecules that are exemplified as covering the sensory perception of human olfaction (odor quality) [61] should be measured first as a priority. Thereby, the human OR sensor must show that it can indeed detect almost all odors perceived by human olfaction. Then, a lot of basically safe odor molecules (e.g., natural flavors and fragrances, synthetic fragrances, food additives) can be measured by the human OR sensor to obtain an odor matrix to enrich the database. The odor matrix is a quantitative and univocal expression of the binding pattern of the human ORs and odor molecules and is a highly objective description of all odors. In addition to the realization of human olfactory DX, the odor matrix can be used to predict the odor quality, i.e., to accurately predict what a human perceives as an odor. Similarly, it will also be possible to predict physiological activity for humans. Specifically, since the odor quality and bioactivity of various odors that have been accumulated so far are often defined in ambiguous language, it is important to utilize neural networks that link them to the odor matrix.

The first step in odor reconstitution is to find ORs that are preferentially used in human olfaction from the odor matrix database. Then, odor molecules that selectively stimulate limited numbers of ORs (one OR if possible) without exceptional activities (see Section 6) should be found from the odor matrix database to create an “odor molecule library for reconstitution”. When reconstituting an odor of interest, the target odor is measured with a human OR sensor to obtain an odor matrix (including changes in each human OR response over time). The minimum group of odor molecules necessary to reproduce this target odor matrix is selected from “the odor molecule library for reconstitution.” The mixing ratios of each odor molecule are then optimized and sprayed from a diffuser or the like to achieve odor reconstitution. The most significant challenge for human olfactory DX is real-time odor reconstitution, which will have a great impact on the world if it is utilized in video equipment that releases the desired odors on demand.

## Data Availability

Data are available from the authors on request.
